# A New Operation for the Cure of Amaurosis, Impaired Vision, and Shortsightedness

**Published:** 1841-10

**Authors:** 


ART. VII.—*A New Operation for the Cure of Amaurosis, Impaired Vision, and Shortsightedness.* By JAMES J. ADAMS, F.L.S. G.S. &c. —*London*, 1841. 8vo, pp. 50.

THE following brief extracts indicate the nature of the cases termed Amaurosis by Mr. Adams, their alleged pathology, and the mode of cure recommended and practised by him:

"The term *amaurosis*, as here about to be employed, will be used more in accordance with its true meaning, by signifying, simply, a dim or darkened sight, without implying any organic disease of the optic nerve.

"Having thus restricted the term amaurosis to the expression of a symptom, common to many diseases of the eye, namely, dim or darkened sight, a necessity arises for distinguishing, by an additional term, the different cases of impaired vision in accordance with their degree or cause; therefore, as the *cause* of the amaurosis, which will form the subject of the following pages, will be found to depend on muscular action, and not on any disease or change of structure in the nerve, I propose to distinguish it from all the other forms of blindness by the term *Muscular Amaurosis*." (p. 11.)

"The simple and uncomplicated forms of Muscular Amaurosis, which it is, particularly, the intention of this paper to enquire into, will be characterized, on the one hand, by a perfect transparency of the humours of the eye, and a healthy condition of all its structures; on the other hand, by the absence of those signs which render the presence of permanent diseases or changes in the brain or optic nerve evident and certain." (p. 12.)

"I am of opinion that the peculiar form of amaurosis, here designated by me as *Muscular Amaurosis*, depends on the bending or partial folding, and compression of the optic nerve, caused by the shortening and thickening of the recti muscles during a state of morbid contraction, which further may be attributed to an affection of the third and sixth nerve, probably at or near to their origins." (p. 42.)

"The operation which I propose for the cure of muscular amaurosis consists in the division and *the extensive separation* of one, two, or more of recti muscles: of which, be it observed, the separation must be *equal* and *even* in each instance. The mode by which I perform this operation I have made peculiarly my own, by adapting a particular set of instruments to it, and by requiring, for its perfect success, a very extensive separation of the muscle, not only from the sclerotica, but from the cellular tissue and conjunctiva which lies in front of it." (p. 25.)

The general statements are illustrated and supported by fourteen cases supposed by Mr. Adams to be examples of the affection of which he writes, and which, generally speaking, are of that kind familiarly known to surgeons not as amaurosis but as nervous asthenic affections of the retina, and originating for the most part in over-exertion of the organ. For nine of these cases no operation was performed, and no special treatment adopted. In the remaining five the operation was performed and, according to the statements of the patients as here recorded, with more or less benefit in all.

The following are some of the remarks which are naturally suggested by the contents of this pamphlet:

1. The theory of the disease maintained by Mr. Adams is not only not supported by the known anatomy and physiology of the eye, but is much at variance with these.

2. In the nine cases not operated on, there is not a shadow of evidence that the symptoms depended on the alleged cause; and in the cases operated on the only evidence rests on the subsequent improvement of vision: and even admitting the improvement to be as here stated, it may be accounted for as plausibly on other grounds as by Mr. Adams's theory.

3. But supposing the theory to be false, if it is true that Mr. Adams by his operation has put it in our power to cure or greatly relieve a numerous class of cases often untractable by other means (although these cases are not Amaurosis) it must be admitted that he has done much more for the healing art than if he had excogitated the most consistent theory. Has he done so? We must be allowed to doubt this for the following reasons:

1st, Because some of the cases detailed by Mr. Adams do not seem to us so strongly in favour of immediate benefit from the operation, as they are supposed to be; 2dly, because the period of alleged improvement as here recorded, that is, the time since the operation, is in all the cases too short, being in the longest of the five cases barely three months, in one six or seven weeks, and in two only eight or nine days; 3dly, because much of the evidence in favour of the improvement rests on the testimony of the patients, and because we know that some of the patients have given to others *different* evidence from that given to Mr. Adams.

These we think are sufficient reasons for the qualified opinion we now entertain of the merits of Mr. Adams's operation: we are, however, quite open to conviction on further evidence, and shall be delighted to acknowledge these merits to be of a very different kind when he has favoured us with proofs that appear to us satisfactory.

We cannot, in justice to the office we fill or to Mr. Adams's own character, close this notice without reference to a subject rather of professional etiquette than of literary or scientific criticism. What does Mr. Adams mean by appending to a surgical treatise the following memorandum?

"N.B. Communications, intended for the Author, are particularly requested to be directed to Mr. JAMES J. ADAMS, No. 27, NEW BROAD St., City, there being another Surgeon of the name of Adams in the Street."

What communications does Mr. Adams expect? Is he so little sensible of his own merits as a surgeon, that he must guard against being mistaken by the passer-by? We are disposed to regard this unfortunate notice as a mere piece of bad taste thoughtlessly committed; but all Mr. Adams's readers may not be so charitably disposed, and we notice it here to warn him to guard in future against the painful mistakes such an advertisement is calculated to occasion in the minds of his brethren.

